# Nanomaterials-Based Novel Immune Strategies in Clinical Translation for Cancer Therapy

**DOI:** 10.3390/molecules28031216

**Published:** 2023-01-26

**Authors:** Shadma Wahab, Mohammed Ghazwani, Umme Hani, Abdulrahim R. Hakami, Abdulrahman A. Almehizia, Wasim Ahmad, Mohammad Zaki Ahmad, Prawez Alam, Sivakumar Annadurai

**Affiliations:** 1 Department of Pharmacognosy, College of Pharmacy, King Khalid University, Abha 62529, Saudi Arabia; 2 Department of Pharmaceutics, College of Pharmacy, King Khalid University, Abha 62529, Saudi Arabia; 3 Department of Clinical Laboratory Sciences, College of Applied Medical Sciences, King Khalid University, Abha 61481, Saudi Arabia; 4 Department of Pharmaceutical Chemistry, Drug Exploration and Development Chair (DEDC), College of Pharmacy, King Saud University, Riyadh 11451, Saudi Arabia; 5 Department of Pharmacy, Mohammed Al-Mana College for Medical Sciences, Dammam 34222, Saudi Arabia; 6 Department of Pharmaceutics, College of Pharmacy, Najran University, Najran 11001, Saudi Arabia; 7 Department of Pharmacognosy, College of Pharmacy, Prince Sattam Bin Abdulaziz University, Al-Kharj 11942, Saudi Arabia

**Keywords:** immunomodulation, cancer, nanomaterials, physicochemical parameters

## Abstract

Immunotherapy shows a lot of promise for addressing the problems with traditional cancer treatments. Researchers and clinicians are working to create innovative immunological techniques for cancer detection and treatment that are more selective and have lower toxicity. An emerging field in cancer therapy, immunomodulation offers patients an alternate approach to treating cancer. These therapies use the host’s natural defensive systems to identify and remove malignant cells in a targeted manner. Cancer treatment is now undergoing somewhat of a revolution due to recent developments in nanotechnology. Diverse nanomaterials (NMs) have been employed to overcome the limits of conventional anti-cancer treatments such as cytotoxic, surgery, radiation, and chemotherapy. Aside from that, NMs could interact with live cells and influence immune responses. In contrast, unexpected adverse effects such as necrosis, hypersensitivity, and inflammation might result from the immune system (IS)’s interaction with NMs. Therefore, to ensure the efficacy of immunomodulatory nanomaterials, it is essential to have a comprehensive understanding of the intricate interplay that exists between the IS and NMs. This review intends to present an overview of the current achievements, challenges, and improvements in using immunomodulatory nanomaterials (iNMs) for cancer therapy, with an emphasis on elucidating the mechanisms involved in the interaction between NMs and the immune system of the host.

## 1. Introduction

Cancer evolves and spreads due to the inability of the host immune system (IS), which is one of the reasons to identify tumor antigens and mount successful protection against them. During the process of immune surveillance, the IS of the host is accountable for identifying and eliminating cancer cells in the body [1,2,3]. Tumor cells evade immunosurveillance by dodging the anti-tumor mechanisms of the IS [4,5]. The anti-cancer capabilities of immune cells that have infiltrated a tumor may be suppressed by signals generated by the tumor itself that are present in the tumor microenvironment. They are also altered to hasten the development and growth of tumors [6,7]. Therefore, the IS performs a contradictory function by contributing to developing an immunosuppressive microenvironment that promotes cancer advancement [2,8]. Immunosuppression is the diminished capacity of the body to generate an immune response due to a lack of immune cells such as T and B lymphocytes [1]. Chemotherapy is a standard treatment for various tumors; however, owing to a reduction in the number of immune cells such as T and B lymphocytes [5] and dendritic cells (DCs), this therapy often results in immunosuppression [9,10]. Nevertheless, it has been shown that many chemotherapy agents affect immunity by preventing the bone marrow from producing blood cells, leading to a sharp drop in the body’s total cell count and compromised defenses [4,5,11].

Immunomodulation optimizes the immune response by inhibiting it, as in treating autoimmune illnesses, or stimulating it, as in cancer immunotherapy or vaccination. Immunotherapy, which uses the body’s IS to fight cancer, has received much attention and is now a common way to treat cancer. However, immunology is only one of several medical subfields where the potential of nanotechnology has been investigated. The nanomaterials (NMs)-based transporter is a critical mechanism to accomplish these aims since nanocarriers may be customized to give a variety of treatments to modify the IS [11,12]. The effectiveness of NMs depends on their direct involvement in the delivery of medicines and improved targeting of diseased immune cells and tissues. However, little attention has been paid to the impact of non-carriers on the control of the IS [13,14]. To find a solution to this problem, researchers have been looking at a wide variety of nanomaterials that may either directly exploit the IS due to the composition of the nanomaterials themselves, or indirectly exploit the IS by acting as intact active carriers. This review examines the functioning of the IS in cancer and the relationship between NMs and the IS to locate and eliminate cancer cells actively or passively. The current evolutions in NMs and their applications in cancer treatment are presented, with a primary emphasis on explaining their mechanism of action to understand the interactions between nanomaterials and the IS.

## 2. Cancer and the Role of the Immune System

Understanding carcinogenesis and the intricate relationship between the IS and the host has been the driving force behind many fundamental improvements in cancer treatment over the last several decades. The standard treatment for cancer includes surgery, radiotherapy, and cytotoxic drugs that simultaneously target many tumor cell types [15]. The IS is the body’s natural defense against pathogens and foreign materials. Considerable research has been conducted to either manipulate the IS to prevent allergy and autoimmune reactions or harness the IS to cure cancer and infectious illnesses [16,17]. These defensive systems affect a wide variety of organs, as well as cells, tissues, and chemical mediators of IS. They are based on the capacity to discern between “self” and “non-self” substances. The IS’s two main building blocks, adaptive and innate immunity, coordinate the body’s defenses against infections and transformed cells. As a result, the IS’s actions and immune cells serve as a protective barrier against invasive diseases and cancer to restore and maintain homeostasis [18]. The first line of defense of the IS is innate immunity, which utilizes cells and molecules already in place to protect against the disease within an hour of the first encounter with the pathogen. The IS’s first line of defense comprises mucosal linings, skin, cilia, and many other physical barriers.

Non-specific innate immunity serves as the initial line of protection. Inflammation is triggered when pathogen-associated molecular patterns from bacteria and viruses are identified by pattern recognition receptors (PRRs) on immune cells. Nucleotide oligomerization domain-like receptors are cytoplasmic PRRs, whereas Toll-like receptors (TLRs) are cell membrane-bound PRRs. Neutrophils, dendritic cells (DC), natural killer cells, mast cells, and macrophages are a few of the cell types that make up innate immunity. During NMs’ uptake by these immune cells, NMs may induce innate immunity by activating the inflammasome, a multiprotein complex that contains PRRs. In response to a trigger, a group of NLRP3 proteins, procaspase-1, and an adaptor protein involved in apoptosis, the speck-like protein, combine to create the NLRP3 inflammasome [19,20]. The pro-caspase-1 enzyme cleaves during this traditional activation method, generating active caspase-1. It breaks down pro-IL-18 and pro-IL-1β precursor cytokines to produce IL-18 and IL-1β, proinflammatory cytokines. The NLRP3 gene was shown to be sufficiently activated by potassium ion efflux, commonly known as the reduction of cytosolic potassium ions [21,22].

Interactions between the IS and the tumor itself play a significant part in the susceptibility or invulnerability of cancer cells to the activity of anti-cancer drugs. On the other hand, the activation of the IS in healthy tissues after treatment with chemotherapy or radiation is connected to both immediate and long-lasting repercussions, including inflammation and fibrosis. Some immune responses may boost toxicity in normal tissue and lessen the effectiveness of anti-cancer treatments. Conversely, manipulating immune responses may increase anti-tumor therapy’s efficacy and reduce the toxicity experienced by normal tissue [23]. Chemotherapeutic and immunotherapeutic medications influence all tissues. Immunotherapy and chemotherapy are due to severe responses to the IS [24,25,26]. The activity of each kind of immune cell, which includes both immunosuppressive and inflammatory cells, can regulate the activity of other cell types [27,28]. The development of immunomodulatory medicines designed to restore the host’s anti-tumor immune response has led to the identification of new therapeutic targets. Therefore, it is necessary to understand the complex interactions between the environment of the tumor, known cancer-causing agents, the IS, and traditional cancer treatments to make headway against the disease’s refractory and recurrent complications and morbidity. This review highlights the complex milieu of cancer immune responses, pointing out the potential nanomaterials-mediated immunomodulation for cancer therapeutics.

**Figure 1:**
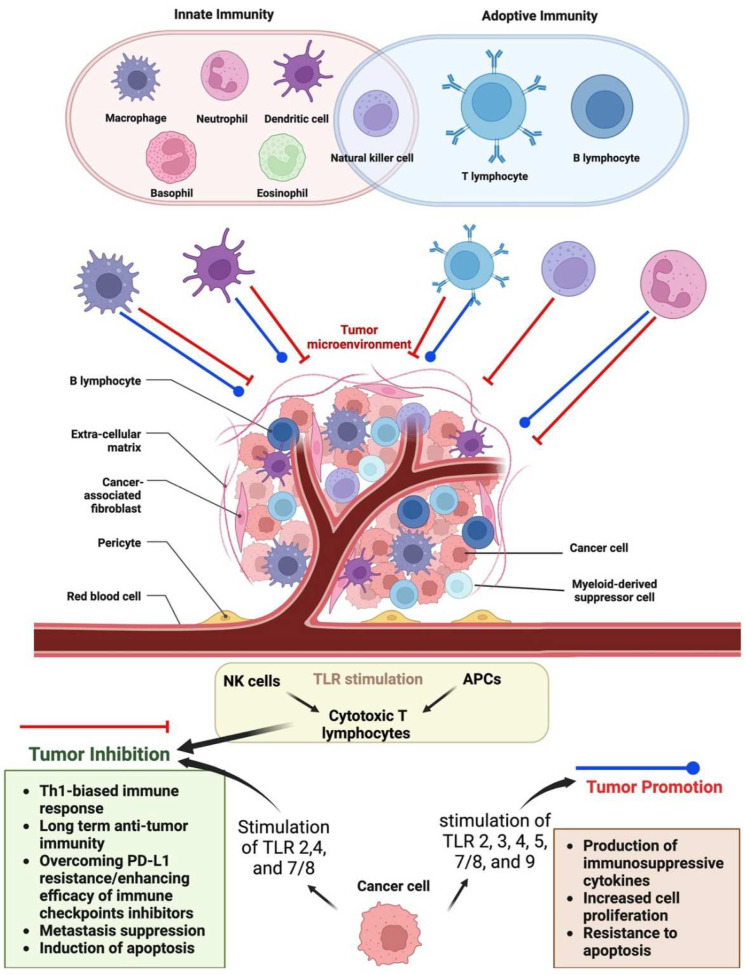
The innate/adaptive immunity and interaction of immune cells in a tumor with the mechanisms of inhibition and promotion.

Immune cells exhibit varied behavior governed by intricate interactions within the microenvironment of tumors. Although it is generally accepted that an immune response specific to the tumor inhibits cancer progression, it is now known that some forms of inflammation linked to tumors might have the opposite effect. Macrophages, neutrophils, mast cells, and natural killer cells are the cells associated with an adaptive immune response, i.e., T and B lymphocytes. Although it is generally believed that an immune response specific to the tumor suppresses cancer progression, it is evident that some forms of inflammation linked with tumors may potentially have the opposite effect. For example, the generation of immunosuppressive cytokines, an increase in cell proliferation, and resistance to apoptosis can all contribute to the growth of a tumor when TLRs 2,4, and 7/8 are stimulated. Contrarily, tumor inhibition can occur via many mechanisms when the stimulation of TLR 2,3,4,5,7/8, and 9 is coupled with chemo- or immunotherapy. In addition, the activation of TLRs on NK cells and APCs (such as DCs and macrophages) might lead to the production of cytotoxic T lymphocytes, which further suppresses the growth of tumors [29]. The innate/adaptive immunity and the interaction of immune cells in a tumor with the mechanisms of inhibition and promotion have been shown in Figure 1.

## 3. Nanomaterials for Indirect and Direct Immunomodulation

Nanotechnology has been looked at in many different areas of medicine, including immunology, where its applications are helpful [30]. The IS plays a critical role in the body’s defenses and has a dispersed nature; hence, using its strengths in therapy has long appealed. Immunomodulation is the process of optimizing the immune response, either by inhibiting it, as in treating autoimmune illnesses or by stimulating it, as in cancer immunotherapy or vaccination. To do this, a wide range of NMs have been studied to see if they can directly affect the IS through their make-up or indirectly through intact active carriers. In this section, NMs and drug delivery systems (DDS) that are utilized to influence the immune response are discussed. It outlines several nanoparticle structural kinds and discusses their makeup and interactions with the IS. In recent research, new nanoparticles and more conventional ones were used. The most advanced ones go beyond administering a single ingredient and go towards combination medication delivery systems and formulations sensitive to stimuli.

There is little room for debate about the fact that nanoparticles provide genuine and novel prospects in various sectors, including healthcare and materials research [31]. These particles are tiny enough to access practically all body parts, including organelles and cells, which may develop novel nanomedicine strategies. The use of nanostructures as diagnostic instruments [32], biosensors [33], and carriers for the targeted administration of drugs [34] has a significant amount of untapped potential. In the broadest sense, NMs are structures with at least one dimension decreased to 1–100 nm. The European Medicine Agency and the Food and Drug Administration in the United States came up with this expanded range [35,36]. NMs are distinguished from bulk materials of the same composition by having physicochemical qualities distinct from those of bulk materials, such as the huge surface area-to-mass ratio, ultra-small size, and high reactivity. Compared to their larger counterparts, NPs have more free functional groups accessible for reactions [37]. Because of their subcellular size, the particles that are used may circulate freely throughout the body and traverse any biological barriers that may be present. This causes a buildup in tissues already prone to it or in specific cells [34]. These characteristics of NPs make passive targeting possible by using both their physical properties and the tissues’ features, such as enhanced penetration and retention in malignancy or leaky vasculature. On the other hand, active targeting occurs due to NPs’ surfaces being transformed in response to different markers produced on specific cells and interactions with their functionalized surfaces. Both approaches of targeted administration increase the amount of the drug at the intended site of action, while decreasing the concentration of that drug in other areas of the body. Consequently, the drug’s effectiveness is enhanced, its adverse side effects are reduced, and the dose needed is decreased. People are more likely to take their medications exactly as directed, which may have therapeutic benefits [38].

**Figure 2:**
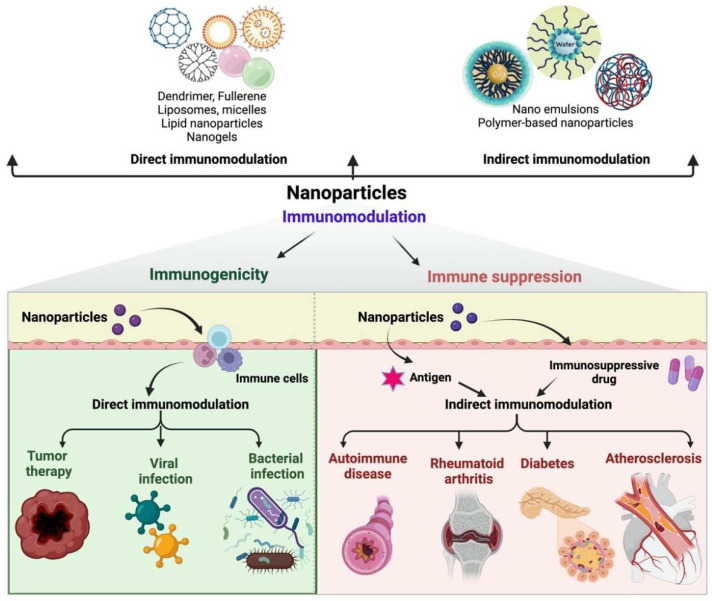
Nanomaterials for indirect and direct immunomodulation. NMs serve as a carrier, and by releasing an immunosuppressive medication or antigen, the immune response is modulated indirectly. Interaction between NMs and IS is a form of direct immunomodulation.

NMs may serve as modulators by directly interacting with the IS, altering the immunological response. In all other circumstances, a modulator may be carried by NPs that have drugs loaded onto them, with the payload being the only element intended to modify the IS’s response. NPs only operate as intact delivery vehicles while modulating IS in a roundabout way. Monitoring immunological reactions to different stimuli is challenging due to the complexity and widespread nature of the IS. Everyone does not entirely understand the interactions between the IS and NMs. The biodistribution of nanoparticles in vivo is a significant challenge in nanomedicine that must be overcome if immunotherapy and vaccination are to be successful. It is vital to know the distribution of NMs in vivo to control the immune response using NMs successfully. Even though many studies detail NP research in vivo [39], there has only been a little research conducted on how polymeric NPs are distributed across the immune system cells. Much research using model nanocarriers was conducted to comprehend how NMs are distributed in the body. Studies employing proteins encapsulated in poly (lactic-co-glycolic acid) PLA NPs have shown the activation of cytotoxic CD8^+^ T cells and the development of significant anti-tumor action [40]. Researchers have shown a connection between the biodistribution of AuNPs and several immune system cell subtypes. These cell types include granulocytes, T cells, and DCs [41,42]. There have also been reports of polystyrene nanoparticles’ immunological imprints and differential uptake by B cells, DCs, and macrophages [43]. The direct connections between NPs and IS immunity were recently the subject of more thorough research [44]. When employing NMs as nanocarriers, the objective is to lessen the number of direct contacts between the IS and the carrier while indirectly influencing the IS due to the transported substance. To accomplish this goal, the surface will need to be modified appropriately. Several strategies have been developed to reduce the direct immunomodulatory impact, including altering the surface of NPs to increase their circulating half-life, actively targeting the formulation, or enhancing its uptake by the chosen set of cells [45]. Nanomaterials for indirect and direct immunomodulation have been shown in Figure 2.

The reticuloendothelial system is the primary organ responsible for phagocytic immune system cell recognition; therefore, NPs that are primarily hydrophobic and charged are promptly opsonized in circulation and may be recognized with greater ease. Conversely, uncharged hydrophilic NPs make for a far less appealing target [46]. Particles of sizes ranging from 100 nm to 6 μm are the ones that set off the phagocytosis process [47,48,49]. The contact angle between the particle being targeted and the phagocytic cell is of critical significance concerning the form of targeted particles. The conditions most favorable for internalization include spherical or ellipsoid particles approaching at a 45° angle [50]. It is also related to how the elasticity of particles affects their internalization. Particles with a higher elasticity have a greater chance of being distorted during the phagocytosis process; as a result, more stiff particles tend to concentrate in phagocytic cells [51]. However, the ultimate interactions between NPs and phagocytic cells are complicated due to the NPs’ charge, hydrophobicity, surface chemistry, shape, size, and elasticity [51,52,53].

The half-life in the bloodstream is reduced by phagocytic clearance; however, surface changes may mitigate this effect. These NPs are called “stealth” NPs. Polyethylene glycol (PEG) makes a hydrophilic corona around NPs to protect them [53]. PEG surface density and molecular weight are two PEG corona parameters influencing the circulation time [46]. These properties influence interactions with the IS, and administering the correct dose avoids the formation of PEG antibodies. A shorter circulatory half-life results from what is called the accelerated blood clearance phenomenon [46,54]. In addition, a recent study discovered that PEG antibodies modify the biodistribution of NPs in the mucosa [55]. Therefore, phagocytic cells are an attractive potential target for the control of immunological responses. These phagocytic cells may be actively targeted to increase the distribution of the substance to phagocytic cells. Phosphatidylserine is an “eat me” signaling molecule used often. One of the components that make up the inner cell wall is called a phospholipid. Phosphatidylserine is displayed on the surface of a cell as a signal for phagocytic cells to scavenge the injured cell if the cell membrane is broken or the cell is undergoing apoptosis [56]. The injection of these liposomes was shown to be effective in reducing inflammation in an in vivo model. The phospholipid of concern was employed as a component of the liposomal bilayer [57]. Other nanomaterials, including polylactic acid nanoparticles (PLGA NPs), have their surfaces modified using phosphatidylserine [58] and CNT [59], to target the compositions of macrophages. This was conducted to target the formulations. In addition to organic molecules, the attachment of tiny functional groups up to 500 daltons in size has been described. These functional groups include alcohols, carboxylic acids, primary amines, sulfhydryls, and anhydrides. Several distinct cell lines, including active and resting macrophages, were tested for the presence of magnetic nanoparticles with functionalized monocrystal surfaces. Even tiny functional groups effectively targeted various cell types and diverse physiological states in macrophages [60].

When a dead cell is merged with an antigen, an immunogenic cell death (ICD) process initiates the adaptive immunological response in the immune host. Dying tumor cells associated with damage-associated molecular patterns such as calreticulin exposure, ATP secretion, ANXA1, and type I interferons (IFNs) expression, and the release of non-histone nuclear protein high-mobility group box 1, altogether promote cell corpses and debris engulfment by antigen-presenting cells, resulting in dendritic cell maturation. Additionally, dendritic cell activation encourages CD4 + and CD8 + T cell priming, triggering cytotoxic T lymphocyte (CTL) and immunogenic T helper 1 (Th1) cell responses. These are the key steps in ICD-induced immune cell realization and immunosuppressive retaliation. Antigens and adjuvants are delivered via nanoparticle-based delivery systems that target lymph nodes that drain tumors [61]. Recent research on NPs for ICD-inducer delivery into tumor cells aims to enhance the immunostimulatory effects, and, subsequently, cancer immunotherapy. The excitation of immunostimulatory cells, cytokines, and chemokines, aided by immunoinhibitory cells and cytokine suppression (IL-4, IL-6, and IL-10), revealed a potential oxaliplatin-mediated ICD in the nano-folox [62]. Studies have shown that ROS is crucial for ICD induction, suggesting that the combined effects of ROS-triggering strategies and NP-based ICD-induction treatments might improve cancer immunotherapy. A systemic delivery platform nanoscale coordination polymer core-shell particle aimed to transport both ROS-triggering agent dihydroartemisinin and chemotherapeutic agent oxaliplatin [63,64].

However, there are few kinetic investigations of polymeric NPs’ cellular biodistribution in the IS. The results of research carried out by Yang and Luo showed that NPs (polystyrene yellow-green, 500 nm) are biodistributed in immunological organs, which implies that they might be helpful in the rations design of formulations. It is crucial for developing immunotherapies based on the targeted administration of NPs to comprehend the kinetics of biodistribution of polymeric NPs in the IS. Blood and bone marrow double-negative cells, splenic dendritic cells (DCs), and monocytes were all evaluated for their phagocytic capacity. It would be helpful to grasp how NPs are distributed throughout the body in vivo to improve or modify immunity [65].

The advent of nanotechnology has opened the possibility of modifying the immune response to either reduce undesirable immune overreactions or adjust interactions between the IS and incredibly deadly substances. In the domain of immunology, NPs are being used as vehicles for the delivery of drugs. In addition, transporters for immunoadjuvants or antigens are also included in the product. They are divided into groups according to the substance’s chemical structure from the vast pool of natural materials already in existence. The non-viral, physiologically applicable drug delivery nanosystems are the focus of this review. In the field of life sciences, scientists are actively using a wide variety of nanomaterials for various applications. Table 1 provides an overview of several immunotherapies that use nanomaterials.

## 4. Physicochemical Properties of Nanomaterials and Their Impact on the Immune System

In biomedical research, nanomaterials consisting of polymers, lipids, and inorganic materials have several potential applications [81,82,83,84,85]. The immunological homeostasis is interfered with by a wide range of NMs’ physicochemical features, which increase the unpredictability of nanomedicine in vivo [86]. Nanocarriers, which can be changed to deliver different therapies to tune the IS, are a crucial way to reach these goals [11,12]. Their effectiveness has been chiefly ascribed to the direct action of treatments that have been delivered and to improved targeting of immune cells or diseased tissues, with the impact of nanocarriers on immunological regulation receiving little attention [13]. The dominant factors affecting the outcome of immune modulation are the shape, size, charge, rigidity, and surface chemical composition of NMs. These criteria are taken into consideration while reviewing the immunomodulatory abilities of NMs. Even though the results of immunological modulation are usually the combined impacts of several parameters, examining the effects of specific components may provide insights into how best to tune the physicochemical features of NMs for immunomodulation. The NM-induced immunological responses shown in Table 2 indicate a wide range of NM physicochemical parameters, including shape, size, charge, rigidity, and surface composition. The ability of nanotechnology to modulate the IS opens the possibility of treating a wide variety of ailments [87,88]. However, the outcome is negatively impacted by the different NMs’ ability to provoke uncontrolled immunological responses. The importance of natural molecules in immune regulation was further established by the phenomenal success of two mRNA COVID-19 vaccines developed by Moderna and Pfizer-BioNTech [89,90,91]. This review discusses the immunomodulatory effects of NMs concerning these variables.

### 4.1. Size-Dependent Immunomodulation of NMs

NMs’ size is a significant consideration for nanomedicines to modulate the IS [131,132]. Their interstitial mobility and biodistribution in vivo are controlled by the NM size of the particles [133,134,135,136]. The primary difficulty associated with their systemic administration is the elimination of NMs from the body during normal blood circulation [137]. In a study, researchers examined the polymeric NPs to precisely target and control the co-delivery of medicine with various physicochemical properties to cancer cells. They co-delivered the docetaxel and cisplatin to prostate cancer cells with synergistic cytotoxicity. The results show that the kidney efficiently clears NMs smaller than 5 nm. When compared to NMs larger than this size range, those with a size between 50 and 100 nm often have a greater lifetime in circulation. In vitro toxicities showed that the targeted dual-drug combination NPs were better than NPs with a single drug or NPs that were not targeted [138]. PLLA-b-PEG polymersomes with a single crystal-like crystalline structure and an average diameter of around 200 nm have very long blood circulation times, with 47% of the injected NP still present in the blood 24 h after injection [139].

The health risks of AgNP are likely to increase with the increasing number of NP-containing products and have shown an adverse reaction in various cell lines. The amount of exposure and a fixed time point measure the results of a toxicology test. The kinetics of NP uptake and the time-dependent intracellular concentration are not commonly considered. The initial line of defense against foreign invaders, such as NPs, is provided by macrophages. The macrophage response to NPs is crucial in determining whether the NPs are harmful. However, investigations on the uptake of nanometer-sized particles and macrophage-like cells are severely lacking. The research was conducted on which uptake rates were measured over 24 h for three different sizes of AgNPs (20, 50, and 75 nm) in a medium containing and without fetal calf serum. The non-toxic concentration of 10 ng Ag/mL for monocytic THP-1 cells was used for this study. This concentration represents a realistic exposure level for short-term exposures. The uptake of silver was more significant in a medium that did not include fetal calf serum, and the results demonstrated that the uptake increased with decreasing NP sizes, both in terms of the NP mass and the NP number. This study’s findings indicate that the uptake rate of NPs by macrophages varies depending on the size of the NPs [140]. The nanoparticle shape influences antibody and cytokine production [141].

### 4.2. Immunomodulation of NMs by Shape

Currently, many medicines have been authorized for use or are undergoing clinical studies, and it is anticipated that nanotechnology will soon be included in many commercial items [142,143]. The shape of the NMs is another critical element that plays a role in their immunomodulation and size [133,134,135]. The research was conducted to determine the precise impact of shape on the biodistribution of predetermined AuNPs after intravenous delivery in mice. They integrated quantitative data derived by inductively coupled plasma mass spectrometry with observational results from histochemistry. Researchers utilized healthy mice that could mount an immunological response since the bio–nano interaction involves the IS. It has been proven that the form of the NM has a role in determining whether they can avoid being removed by the reticuloendothelial system. In filter organs, the kinetics of AuNP buildup and excretion are significantly influenced by shape [136]. It is known that µm-sized flexible filaments are less absorbed by macrophages than spheres and short filomicelles, due to their expanded conformation under blood flow, lengthening the blood circulation of long filaments [144]. The varied ways NMs interact with cells are the root cause of the disparity in their biodistribution. The NM form partially governs the interactions. As a result, NM shape may be manipulated to control tissue-targeting and immunological regulation [145,146,147]. However, the ultimate interactions between NPs and phagocytic cells are complicated due to the NPs’ charge, hydrophobicity, surface chemistry, shape, size, and elasticity [51,52,53].

### 4.3. Immunomodulation of NMs by Rigidity

Immune cells can detect and react to biophysical stimuli ranging from dynamic stresses to spatial characteristics throughout their formation, activation, differentiation, and expansion. These biophysical signals control the functions of immune cells, such as the release of leukocytes, the selection and activation of T cells, and the polarization of macrophages. In addition, integrins and focal adhesion complexes play a significant role in the contact between the cell and the matrix. Ion channels are another kind of mechanosensors. These channels gate soluble ions such as Na^+^, K^+^, and Ca^2+^ [148]. The function of each cell is governed by the aggregate signals that are received from a variety of immunoreceptors. The expression and activity of immunoreceptors are contingent on the cell’s current development stage and the surrounding environment [149]. Recent research has shed light on the existence of mechanical force on several different immunoreceptor–ligand pairs, as well as the significant role that force plays in regulating the interaction and function of these couples. The pharmacokinetics of NM and the effectiveness of intracellular drug delivery are both affected by the mechanical forces created during interactions between NMs and cells [150,151]. The effectiveness of cancer vaccines based on peptides is limited in people, despite the enormous promise these vaccines provide. Recent advancements have heralded a new age of personalized immunotherapy using patient-specific neoantigens in tumor exome sequencing. Yet, there is still a lack of a general strategy for inducing potent CD8α+ cytotoxic T-lymphocyte responses. In addition, vaccination with many epitopes led to broad-spectrum T cell responses, effectively preventing tumor development. When paired with treatment targeting anti-PD-1 and anti-CTLA-4 receptors, nanodiscs successfully eradicated both the MC-38 and B16F10 tumors. These results indicate a broad method for tailored nanomedicine and provide a novel, very effective approach to the treatment of cancer immunotherapy [152]. When developing delivery systems for vaccines and immunotherapies, it is essential to consider the relevance of biomechanics and rigidity. It is possible to alter the rigidity of NMs to direct them to the lymphoid organs and enhance their biodistribution. As a result, soft NMs could have an advantage in homing to lymph nodes, which would boost the administration of immunomodulators.

### 4.4. Immunomodulation of NMs by Surface Charge

Another physicochemical characteristic influencing NM fate is surface charge [153,154,155]. Most DDS are colloidal and may have positive or neutral surface charges. Researchers attempted to control the surface charge of drug carriers through the change of surface chemistry or other techniques, including introducing positive or negative charges to the surface of DDS. The surface charge may be affected in several different ways. A neutral surface charge increases circulation time and inhibits the adsorption of plasma proteins to particle surfaces. On the other hand, a positive surface charge may facilitate improved contact with the cellular membrane and internalization; nevertheless, this may also have a toxic effect on the cells. A negative charge not only has a less harmful impact, but also decreases the cell’s ability to adsorb particles [156,157]. It may be possible to create an appropriate carrier for DDS by imparting a positive charge on the surface of particles, combining with incorporating stealthy materials (such as PEG) or targeting agents [154]. The charge that is present on the surface of the DDS has the potential to increase cellular uptake. This is particularly true if the charge is positive, as this may interact with the negative charge on the cell membrane to stimulate adsorption.

The contact between NMs and immune cells may be mediated by proteins adsorbed on their positively charged surfaces. There have also been contradictory reports about the influence of charge on NM internalization by immune cells [158]. Aside from cell uptake, the NM surface charge influences NM biodistribution and immune cell activation in vivo [103,159,160,161]. Researchers intravenously injected mice with functionalized gold nanoparticles and employed quantitative imaging based on laser ablation inductively coupled plasma mass spectrometry to display the surface charge changes in the suborgan distributions of NPs in the liver, kidney, and spleen. The kidney images show that positively charged nanoparticles accumulate extensively in the glomeruli, which is the initial stage in the filtering process for the nephron. It suggests that the kidney may filter these nanoparticles at a different rate than the neutral or negatively charged nanoparticles that the kidney would filter. The red pulp of the spleen is where researchers observed a significant accumulation of nanoparticles with both positive and negative charges. However, unlike positively or negatively charged nanoparticles, uncharged particles build up more in the spleen’s white pulp and peripheral zone. In addition, the likelihood of these uncharged nanoparticles being identified as connected with Kupffer cells in the liver is increased. Nanoparticles with a positive charge build up in the liver hepatocytes, while nanoparticles with a negative charge have a more widespread distribution throughout the liver. These observations, taken together, point to the possibility that neutral nanoparticles with cores measuring 2 nm may interact with the IS to a greater extent than charged nanoparticles. This finding highlights the importance of determining the suborgan distributions of nanomaterials for applications involving delivery and imaging [162]. The spleen is the body’s largest secondary immune organ. It is vital to start immunological responses to blood-borne antigens and filter the blood of foreign substances and damaged or old red blood cells. The red and white pulp, the two primary compartments of the spleen, are responsible for performing these duties. The red and white pulps are quite distinct in their vascular organization, architecture, and cellular makeup. The charged AuNPs exhibited a substantially lower concentration in the spleen’s white pulp and marginal zone than the neutral AuNPs [163].

**Figure 3:**
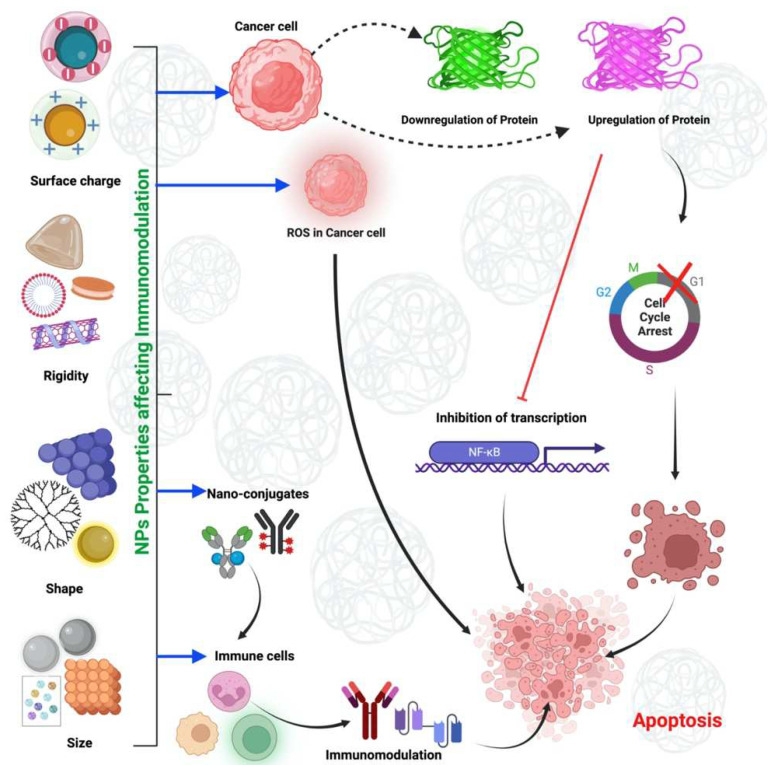
Physicochemical properties of nanomaterials and mechanisms of cell death in cancer induced by nanoparticles.

Furthermore, neutral AuNPs were found in Kupffer cells in the liver. Researchers have shown that DCs can be precisely and effectively targeted in vivo by giving RNA-lipoplexes based on well-known lipid carriers through an IV. This is done by adjusting the net charge of the particles in the best way possible without adding molecular ligands to the particles. RNA-LPX (RNA-lipoplexes) represent a universally applicable vaccine class for systemic DC targeting and synchronized induction of both highly potent adaptive as well as type-I-IFN-mediated innate immune mechanisms for cancer immunotherapy [102]. Gene editing using CRISPR–Cas and protein replacement therapy based on messenger RNA promise to efficiently cure disease-causing mutations that may arise from various cell types. A method is known as selective organ targeting (SORT), in which several types of lipid nanoparticles are methodically built to specifically edit extrahepatic tissues by incorporating an additional SORT molecule. By changing the composition of the permanently charged lipids included in the lipid nanoparticles (LNPs), it was possible to target specific organs in mice after the systemic injection of mRNA-delivery LNPs [159]. These results suggested that NMs with neutral or low negative charges may be more efficient in targeting DCs and macrophages in the spleen than their positively charged counterparts. The ability of NMs to adsorb proteins is highly dependent on their surface charge. Further research to identify corona proteins that mediate APC targeting may aid in developing NMs for effective APC targeting. The physicochemical properties of nanomaterials and mechanisms of cell death in cancer induced by nanoparticles have been illustrated in Figure 3.

Differently shaped nanocarriers have their spatial characteristics and some unique benefits and drawbacks. Although NP-based nanomedicine has significantly contributed to cancer immunotherapy, more research is needed to understand the disadvantages of NP-mediated immunogenicity, non-targeted cellular uptake and cytotoxicity, and the precise interactions between NPs and the IS. Furthermore, translating NP-based nanomedicine to clinical applications is difficult due to the scarcity of evidence on the immune system’s role in tumor genesis and progression. Because of the intricacy of innate and adaptive immunity, determining the impact of one component’s depletion or suppression on the entire immune network is difficult. Furthermore, different tumors have heterogeneous structures, making it difficult to predict how they respond to NP-based nanomedicine. Consequently, developing suitable NP platforms for distinct cancers is crucial and difficult [164]. Current toxicity studies for NPs are in their infancy due to their complex physiochemical characteristics and possible interactions with biological components already present in the body. Neither their ultimate structural shape nor their safety after therapeutic administration is known.

## 5. Advantages and Disadvantages of the Different Types of Nanocarriers

Nanodrugs may enhance many pharmacological characteristics of traditional (or ”free”) drugs [165]. NP systems’ primary properties are the Zeta potential, particle size, and size distribution. NPs are distinguished from bulk materials (on the microscale) of the same composition by their ultra-small size, enormous surface area-to-mass ratio, and high reactivity. NPs may encapsulate and transport medications that are not easily soluble when used as therapeutic carriers [166]. These features are often linked to highly desired attributes (electrical, mechanical, and chemical) for specialized medicinal purposes. Still, they may also be the primary determinants defining their potentially harmful consequences on human health [167]. A comparison of various nanoparticles has been exhibited in Table 3. To better understand how nanostructures interact with biological systems, different international scientific societies have emphasized the significance of developing nanotoxicology, a key subdiscipline of nanotechnology. This discipline focuses on elucidating the connections between nanostructures’ physical and chemical properties and the induction of toxic biological responses [167]. Nanodrugs can enter the body via main routes such as lung, subcutaneous, intraperitoneal, intravenous, and oral. The nanomaterials may be toxic at these phases through many mechanisms, such as inflammatory and pro-oxidant activities. The toxicity profiling of NMs has been a highly demanded research area worldwide in recent times. Natural NMs have been a part of the ecosystem for a long time and contain various processes that make them less toxic to living things. Research breakthroughs have shown some immediate hazardous impacts of nanosized particles in biological systems. Emerging NPs, such as viral NPs and nanozymes, should undergo thorough cytotoxicity experiments to determine safe dose levels and application procedures. The success of some nanoparticle-based medications, such as the COVID-19 mRNA vaccines, has generated interest among the public and scientific community regarding their potential application in the treatment of a variety of other diseases, including discussions about the development of a future cancer vaccine [168]. A vaccination for cancer is different from one for an infectious illness. Cancer vaccines may need to use a variety of approaches to overcome treatment resistance. For example, a vaccination using nanoparticles must be designed differently for injection into the blood instead of the muscle. Although the area of nanomedicine has made significant strides in moving medications or diagnostics from the lab and into the clinic, there is still a long way to go. Learning from past successes and failures can help researchers develop breakthroughs that allow nanomedicine to live up to its promise. The benefits and drawbacks of the various nanomaterials have been listed in Table 3.

## 6. Nanomaterial-Based Immunotherapy

Cancer immunotherapy has received much attention recently due to its distinct features and consequences that no other cancer therapies can match. Immunotherapy for cancer may systematically target both primary and secondary tumor metastases. Nanotechnologies have opened up a new direction for research and development, allowing for the efficient delivery of drugs to specific areas of the body and the active targeting of particular cell populations, such as tumor cells or subsets of immune cells [172]. NMs have been employed extensively in research on cancer immunotherapy because of their unique benefits. NMs’ advancements in cancer immunotherapy, alone or in conjunction with other therapies, include dendritic cell (DC)-targeted delivery systems, self-adjuvants, combination therapy, and engineered APCs [173]. In addition, to improve the response, co-delivery and targeting of nanocarriers are appealing options [174,175,176,177]. Due to their outstanding physicochemical characteristics, which include size, shape, and surface features and result in preferable biological interactions, nanoparticles are a frequently utilized nanomedicine platform in cancer immunotherapy. Furthermore, cancer immunotherapy has excellent potential for using tailored nanostructural materials such as nano-emulsions, nanotubes, and NPs [164].

NPs’ essential and physiochemical characteristics are influenced by many cancer treatment modalities, including chemotherapeutics, nucleic acid-based therapies, photothermal therapy, and photodynamic agents [178]. Antigen-presenting cells may take up nanoparticles to facilitate the cytosolic transport of encapsulated antigens and adjuvants [164]. They may be divided into numerous categories. The NP-based immunotherapy termed ARAC (Antigen Release Agent and Checkpoint Inhibitor) is designed to enhance the efficacy of PD-L1 inhibitors. PLK1(polo-like kinase 1) inhibition increases the expression of PD-L1 in cancer cells, reducing cytotoxic T lymphocytes’ effectiveness. PLK1 inhibition and cancer immunosuppression support the use of the PD-L1 immune checkpoint blockade in conjunction with PLK1 inhibitors as a possible therapeutic approach [179]. Nanoparticles can modulate innate immune cells, including monocytes, NK cells, TAMs, neutrophils, DCs, and MDSCs. For instance, TAMs can act as antigen-presenting cells and produce different solubility to interact with other immune cells. In addition, they play a crucial role in cancer immunotherapy [180,181]. Nanocarriers may enhance medications’ pharmacokinetic and pharmacological characteristics by improving the drug’s water solubility and stability in circulation. Additionally, they allow for tissue or cell-specific drug administration; reducing drug buildup in the kidneys, liver, and other non-targeted organs; and enhancing the therapeutic effectiveness and drug delivery of a medication cocktail [182,183,184]. The conjugated therapeutic is shielded from deterioration by NPs. Furthermore, nanoparticles facilitate drug uptake by epithelial diffusion, enabling medicine concentration to reach optimum levels quickly. NPs alter medicines’ pharmacokinetic and tissue distribution patterns in cancer cells and boost intracellular efflux [182,183,184]. It has been shown that checkpoint inhibitors given by NPs increase the duration of the response rate of T cell-based immunotherapy [185,186]. NPs can be fine-tuned and functionalized with specific moieties to promote their efficacy in targeting and delivering cargo materials to particular locations. Therefore, it has been concluded that NPs could be used as carriers in cancer immunotherapy.

## 7. Future Perspectives and Conclusions

Nanomaterials’ rapid advancement has given cancer immunotherapy a fresh perspective. Mainly NPs provide several advantages over traditional medication delivery methods. Although immunotherapy has shown promising outcomes in various therapeutic applications, there is still a considerable barrier preventing its widespread use in clinical settings. This barrier comprises a low patient response rate and restricted dosage toxicity. As a result, monotherapy still struggles to provide a positive response or prognosis in many individuals. Additionally, monoclonal antibodies’ cost, preparation, and preservation make them unsuitable for widespread usage. To fix the problems with cancer immunotherapy and make it work better as a treatment, researchers have investigated nanomaterials-based combination therapy as an alternative therapeutic strategy to boost immune responses by controlling the many steps of the cancer immunity cycle. Stable, biocompatible nanomaterials may be modified with active targeting ligands or the EPR (enhanced permeability and retention) effect to increase drug accumulation at tumor locations. The development of nanomaterials for immunotherapy has sparked renewed optimism for improving this treatment’s efficacy. A multipronged approach using nanomaterials, immunotherapy, and other medicines has emerged as the primary area of tumor therapy investigation.

The capacity of NPs to be tweaked and functionalized allows them to be constructed in various sizes, shapes, and capabilities to satisfy individual demands. In addition, the direct or indirect targeted delivery of NPs to tumor tissues exploits the tumor vasculature’s hyperpermeability, bolstering cancer immunotherapy and reducing the toxic effects of anti-cancer drugs. Many in vitro and in vivo studies using NPs in cancer immunotherapy have shown positive results. These include considerable drug protection against degradation, prolonged and controlled intracellular delivery, and the avoidance of multidrug resistance in different kinds of NPs. In other trials, NPs have also proven crucial in combining treatment plans, including chemotherapy, phototherapy, and radiation. Furthermore, these nanoparticles can work with various immunotherapies to improve treatment outcomes by reprogramming the immunosuppressive tumor microenvironment and beginning systemic anti-tumor immune responses. However, the practical implementation of nanomaterial-based combination immunotherapies has a long way to go, even though some outcomes have been produced in the laboratory using these immunotherapies. For instance, in clinical studies, only radiotherapy and several combinations of chemotherapeutic medicines and immunotherapies are being investigated as potential treatments for cancer.

It is now widely accepted that nanomaterial-based immunotherapy has great promise for improving the efficacy of immunotherapy, and that a combination approach based on immunotherapy, nanomaterials, and other medicines is the primary area of tumor therapy research. This review demonstrates that various well-known and new polymeric and inorganic nanoparticles have been fabricated for immunotherapy and synergistic immunotherapy. By reprogramming the immunosuppressive TME and introducing systemic anti-tumor immune responses, nanoparticles may be considered ICD-inducing medications’ modalities that synergize with various immunotherapies to improve treatment results. In clinical studies, only radiotherapy and several combinations of chemotherapeutic medicine and immunotherapies are being investigated as potential treatments for cancer. Nonetheless, a clinical trial assessment of the approach of combining PDT, PTT, or SDT (sonodynamic therapy) with immunotherapy has not been documented; therefore, these combination treatments are still in the early stages of study. Current tumor therapy has progressed from the lab to the clinic, but issues still need to be resolved, such as the low enrichment rate and more significant toxicity of nanomaterials. Cancer immunotherapy will soon be a breakthrough, even though nanomedicine-based immunotherapy and its combination treatment are still in their infancy. Chemists, biologists, and biochemists are all committed to this specialization.

## Figures and Tables

**Table 1:** Different immunotherapies are based on nanomaterials.

Nanomaterial Composition, Carrier	Payloads	Therapy	Properties	Outcomes	References
Lipid-calcium-phosphate NP, liposome-protamine-hyaluronic acid NP	Trp 2 peptide, CpG oligonucleotide, siRNA	Cytokines or chemokines modulation	Down-regulate TGF-β, increase CD8^+^ T cells levels, decrease T_reg_ cells level	Dramatically increase levels of tumor-infiltrating CD8^+^ T cells and decrease T_reg_ cells level	[66]
Lipid-coated protamine DNA complexes	Plasmid DNA encoding TNF-related apoptosis- TRAIL-inducing ligand protein	Cellular modulation	Generate approximately 70% of TAFs as sTRAIL-producing cells	Nanoparticles to modify tumor-associated fibroblasts (TAFs) as an effective strategy to treat desmoplastic cancers	[67]
Porous silicon microparticle	HER2 antigen	DC-based vaccine	High IFN-I and MHC II levels cause CD11c^+^ DC infiltration	PSM stimulate DC-based cancer immunotherapy	[68]
Mannose-modified PLGA nanoplatforms	mannose	Cellular modulation	Deplete M2 TAMs	Lower uptake by regular macrophages	[69]
pH-responsive poly (propylacrylic acid) nanocomplex	α-galactosylceramide (α-GalCer)	Peptide-based vaccine	Improve antigen-specific CD8^+^ T cells responses	Peptide/pPAA nanoplexes are a simple way to increase CD8^+^ T cell responses to peptide antigens	[70]
Au-SGSH nanocomplex	Melanoma antigen (MART1)-encoded DNA vaccine	Nucleic acid-based vaccine	Increase the levels of TNF-α and induce a large amount of CD11c^+^ DC infiltration	Potential use for in vivo DC-targeted genetic immunization against cancer	[71]
PLGA NPs	TLR-4 and PTX agonist	Chemotherapy-induced ICD	Increase APCs and T cells’ activation ability	Increased cancer-fighting power, fewer side effects, and simplified administration	[72]
Multifunctional near-infrared (NIR)-responsive core-shell nanoparticles	gardiquimod	PTT-induced immunotherapy	Activate and increase tumor infiltration of CD8^+^ T and DCs cells, release TAAs	Photothermal immunotherapeutic potential	[73]
Trastuzumab-loaded polyacid nanoparticles	trastuzumab	Tumor-targeted antibody therapy	Signaling transduction and cell-mediated cytotoxicity	PLGA NPs may include TZ and conventional chemotherapeutics	[74]
Poly (ethylene glycol)-block-poly (d,l-lactide) copolymer	CTLA4 small interfering RNA (siRNA)	-	Stimulate T cell activation proliferation by silencing the CTLA4 molecules	Efficient cancer immunotherapy with nanoparticles for melanoma	[75]
RGD-modified single-walled carbon nanotube as artificial tobacco mosaic virus	doxorubicin	Oncolytic virotherapy	Cytomembrane penetration and endoplasmic reticulum disruption cause Ca^2+^ release	Induce robust composite oncolytic processes, including cytomembrane penetration	
MnOx nanospikes	Ovalbumin	Protein-based vaccine	Secretion levels of IL-6 and TNF-α	Effectively inhibit primary/distal tumor growth and tumor metastasis	[76]
Poloxamer 407	Anti-CTLA4 antibodies	Anti-CTLA4 therapy	Decrease systemic antibody levels	Effectively slow down tumor growth, whilst significantly reducing serum anti-CTLA-4 levels	[77]
Fucoidan-dextran-based magnetic nanomedicine	Anti-CD3, anti-CD28, anti-PD-L1	Anti-PD-1/PD-L1 therapy	Decrease the chaotic distribution of anti-PD-L1 and decrease the toxic effects caused by off-target effects.	Potential of integrating anti-PD-L1 and T cell activators	[78]
Photosensitizer (HPPH)-coated αvβ6-targeting peptide-functionalized graphene oxide	Photosensitizer	PDT (photodynamic therapy)-induced ICD	Increase cytotoxic CD8^+^ T lymphocytes infiltration	PDT using GO(HPPH)-PEG-HK may ablate primary tumors	[79]
Fluid lipid bilayer supported by mesoporous silica micro-rods	IL-2, anti-CD28, anti-CD3	ACT	T cell polyclonal growth is increased two to tenfold	APC-ms enables antigen-specific expansion of rare cytotoxic T cell subpopulations	[80]

**Table 2:** Nanomaterial-induced immune responses concerning nanomaterials’ physicochemical properties, such as surface, charge, rigidity, shape, and size.

Nanomaterial Composition	Parameter of Nanomaterial	Model	Immune Cells	Outcomes	References
Poly lactic-co-glycolic acid nanoparticles (MSC-PD-L1+ NPs)	Surface: mesenchymal stem cell membrane	In vivo: intravenous administration	T cells and macrophages	This strategy has been shown to potentially treat various cancers’ immunotherapy-associated irAE in clinical applications.	[92]
Biodegradable polymeric nanoparticles	Surface: natural erythrocyte membranes	In vivo: intravenous administration	Macrophages	After 72 h after receiving the particle injection, the biodistribution analysis found considerable particle retention in the blood.	[93]
Polymeric nanoparticles	Surface: plasma membrane of human platelets	In vivo: intravenous administration	Macrophages	Platelet-mimetic nanoparticles enhanced therapeutic efficacy.	[94]
Plasma polymerization	Surface: hydrophobic, hydrophilic groups	In vitro in vivo: intravenous administration	Macrophages, monocytes, and splenocytes	Surface modifications were made to modulate serum protein adsorption and to achieve the desirable innate immune response to implanted biomaterials and devices.	[95]
Gold nanoparticles (Au NPs)	Surface: inverse phosphocholine lipids	In vivo: intravenous administration	Neutrophils	It has demonstrated the importance of hydrophobicity in IS activation.	[96]
Polymeric nanoparticles	Surface: Poly-ethylene-alt-maleic anhydride	In vivo: intravenous administration	Macrophages, neutrophils, monocytes	These particles might be used in trauma and to treat inflammatory diseases.	[97]
Antigen-capturing nanoparticles (AC-NPs)	Surface: MalAC	In vivo: intratumorally	T cells and DCs	This model might be used for cancer immunotherapy.	[98]
Au NPs	Surface: PEG	In vitro	Human dermal fibroblast	This increases the level of IL-6.	[99]
PLGA	Surface: PEG	In vivo: subcutaneous injection	Neutrophils and DCs	This induced immune tolerance through subcutaneous administration.	[100]
Mesoporous silica	Surface: thiol, amino, and PEG	In vivo: intravenous injection	Macrophages and T cells	This increases TGF-β and T cells.	[101]
Lipoplexes	Charge: negatively	In vivo: intravenous administration	Macrophages and plasmacytoid DCs	This increases the release of IFNα and DC maturation.	[102]
Liposome; polyglutamic acid; chitosan	Charge: negatively	In vitro	Complement and platelet system	This increases complement activation and P-selection.	[103]
Cationic polymers	Charge: positively	In vivo: intraperitoneal injection	Peritoneal macrophages and spleen cells	These increase the level of TNFα, IL-12, and Th1 responses.	[104]
Gold nanoparticles	Charge: positively	In vitro	U937 cells and human lymphoma cell line	These increase the production of IL-6.	[105]
Cationic nanohydrogel	Charge: positively	In vivo: pulmonary immunization	T cells, B cells, and DCs	This increases activated CD4+ T, Germinal center B cells expansion, and activated DCs.	
Lipid nanoparticles	Charge: positively	In vitro In vivo: intravenous administration	Bone marrow-derived dendritic cells, cytotoxic T lymphocytes, and CD11b- cells	These increase ROS generation and CCL2 expression, type I interferon response, Th1 cytokines expression (IL-2, IFNγ, TNFα), and CD8^+^ T cell response.	[106]
Superparamagnetic iron oxide	Charge: positively	In vitro	DCs	This increases the antigen cross-presentation.	[107]
Stiff-nanocapsules	Rigidity: silica	In vitro	RAW264.7 cells, Murine macrophage cell line	These increase cellular uptake.	[108]
PLGA	Rigidity: soft-emulsion droplets	In vivo: subcutaneous vaccination	DCs	This increases DCs and CD86^+^.	[109,110]
Hydrogel	Rigidity:	In vivo: intravenous administration	Spleen cells	This increases the spleen retention.	[111]
Lipid-coated alginate	Rigidity: soft-microparticles with low modulus	In vitro	CD8^+^ T cells	This increases the activated CD8^+^ T cells.	[112]
Polysaccharides	Rigidity: soft-hollow capsules	In vivo: subcutaneous injection	T cells and DCs	These increase the activation of T cells and DCs and increase lymph node targeting.	[113,114]
Polymeric particles	Shape: tetrahedron	In vitro In vivo: intravenous injection	Peripheral blood mononuclear cells, RAW264.7 cells, murine macrophage cell line	These increase the level of IL-6, TNF-α, and IFN response.	[115,116]
Polymeric particles	Shape: spherical	In vitro In vivo: intravenous injection	Neutrophils in normal and encephalomyelitis-inflamed mouse blood	These decrease cellular uptake.	[117]
Polymer capsules	Shape: rod	In vitro	Human monocyte-derived macrophages	These increase the level of IL-8, TNF-α.	[118]
TiO_2_ microparticles	Shape: spike	In vitro	Bone marrow-derived macrophages and dendritic cells	These increase CD40, IL-1β, and IFN-γ.	[119]
Antigen-decorated microparticles	Size: 500 nm diameter	In vivo: intravenous injection	T cells	These increase long-term T cell tolerance, T cell anergy, and regulatory T cell activation.	[120]
Antigen-loaded polylactide particles	Size: 200–600 nm	In vivo: intramuscular injection	J774A.1 cells, murine alveolar macrophage cell line	These decrease Th2-type immune response, IL-4, and MHC-II expression and antibody titers.	[121]
Superparamagnetic iron oxide	Size: 50 nm	In vitro	Human CD8^+^ T cells	The activation of T cells occurs.	[80,122,123]
Small-size silver nanoparticles	Size: 5, 10, and 50 nm	In vitro	Human neutrophil	These increase ROS, NADPH oxidase, and intracellular calcium.	[124]
Polypyrrole nanoparticles	Size: 5 nm	In vitro	J774A.1 cells and murine alveolar macrophage cell line	These increase IL-6, IL-1, and TNF-α.	[125]
Polypyrrole nanoparticles	Size: 20, 40, 60, 80, and 100 nm	In vitro	J774A.1 cells and murine alveolar macrophage cell line	These decrease CD86 and increase CD40, CD80.	[126]
Silica−Titania hollow nanoparticles	Size: 25, 50, 75, 100, and 125 nm	In vitro	J774A.1 cells and murine alveolar macrophage cell line	IL-1, IL-6, and TNF-α. These increase TNF-α, IL-1, and IL-6.	[126]
Graphene oxide	Size:(10–40 μm) and (50–300 μm)	In vitro	Human monocyte-derived macrophages	This increases IL-1β and decreases IL-10.	[127]
Inorganic nanoparticles, particularly iron oxide (IO) and gold (Au)	Size: 4 nm	In vitro	RAW264.7 cells and murine macrophage cell line	These increase M1 polarization and decrease M2 transformation.	[128]
Silver particles	Size: 4 nm	In vitro	U937 cells, human lymphoma cell line	These increase IL-8 and ROS.	[129]
Carbon nanomaterials	Size: 15, 50, 140 nm	In vitro	THP-1 cells, human monocyte cell line	These increase 5 nm: M2 macrophages, 50 nm: M1/M2 macrophage and 140 nm: M1 macrophages.	[130]

**Table 3:** Benefits and drawbacks of the various kinds of nanocarriers.

Types of Nanocarriers	Drawbacks	Benefits
Metallic nanoparticles	Particles’ instability, impurity, biologically harmful, explosion, difficulty in synthesis, toxicity	Biocompatible; strong plasma uptake; and uniformity in size, shape, and branch length. Tuned pharmacokinetics and biodistribution
Dendrimers	Low hydro solubility and high non-specific toxicity, poly(amidoamine) (PAMAM) dendrimers, and PPI dendrimers attributed toward toxic manifestations	Water soluble and biocompatible, good PK behavior, flexibility in conjugation chemistry, and ability to encapsulate and deliver various bioactive agents [169,170]
Polymeric micelles	The low payload of drugs and less stability in an aqueous medium	Biodegradable, self-assembling, and biocompatible. Potential targeting of functional modification, efficient carrier system for hydrophilic drugs, biodegradable
Carbon nanotubes	Poorly soluble in water, not biodegradable, toxicity concerns, poor PK (pharmacokinetics)	Ease of synthesis and conjugation of multiple bioactive agents, large surface area, ability to encapsulate and deliver various types of bioactive agents, protects entrapped drug and provides sustained release [171]
Liposomes	Fewer stables, leakage, and fusion of encapsulated drug/molecules; high production cost; some may be allergic	Targeted to specific cells or tissues, biocompatible, longer duration of circulation, high stability via encapsulation, high efficacy and therapeutic index of drug

## Data Availability

Not applicable.

## Sample Availability

Samples of the compounds are not available from the authors.
